# Knockdown of a novel lincRNA AATBC suppresses proliferation and induces apoptosis in bladder cancer

**DOI:** 10.18632/oncotarget.2833

**Published:** 2014-11-25

**Authors:** Fengjin Zhao, Tianxin Lin, Wang He, Jinli Han, Dingjun Zhu, Kaishun Hu, Weicong Li, Zaosong Zheng, Jian Huang, Wenlian Xie

**Affiliations:** ^1^ Department of Urology, Sun Yat-sen Memorial Hospital, Sun Yat-sen University, Guangzhou, China; ^2^ Lin Bai-xin Research Center, Sun Yat-sen Memorial Hospital, Sun Yat-sen University, Guangzhou, China; ^3^ Guangdong Provincial Key Laboratory of Malignant Tumor Epigenetics and Gene Regulation, Sun Yat-Sen Memorial Hospital, Sun Yat-Sen University, Guangzhou, China

**Keywords:** lincRNA, bladder cancer, proliferation, apoptosis

## Abstract

Long intergenic noncoding RNAs (lincRNAs) play important roles in regulating various biological processes in cancer, including proliferation and apoptosis. However, the roles of lincRNAs in bladder cancer remain elusive. In this study, we identified a novel lincRNA, which we termed AATBC. We found that AATBC was overexpressed in bladder cancer patient tissues and positively correlated with tumor grade and pT stage. We also found that inhibition of AATBC resulted in cell proliferation arrest through G1 cell cycle mediated by cyclin D1, CDK4, p18 and phosphorylated Rb. In addition, inhibition of AATBC induced cell apoptosis through the intrinsic apoptosis signaling pathway, as evidenced by the activation of caspase-9 and caspase-3. The investigation for the signaling pathway revealed that the apoptosis following AATBC knockdown was mediated by activation of phosphorylated JNK and suppression of NRF2. Furthermore, JNK inhibitor SP600125 could attenuate the apoptotic effect achieved by AATBC knockdown, confirming the involvement of JNK signaling in the induced apoptosis. Moreover, mouse xenograft model revealed that knockdown of AATBC led to suppress tumorigenesis *in vivo*. Taken together, our study indicated that AATBC might play a critical role in pro-proliferation and anti-apoptosis in bladder cancer by regulating cell cycle, intrinsic apoptosis signaling, JNK signaling and NRF2. AATBC could be a potential therapeutic target and molecular biomarker for bladder cancer.

## INTRODUCTION

Bladder cancer is a common malignant tumor of genitourinary system[1]. Approximately 70% of the cases are non-muscle invasive bladder cancer (NMIBC) that can be easily resected. However, some of these cases will progress to muscle invasive bladder cancer (MIBC) or metastasis to distant organs and ultimately endanger the lives of patients. Surgery is the main treatment for bladder cancer, but recurrence and metastasis are very common. So far, little is known about the pathogenesis of bladder cancer and no sensitive tumor biomarker has been found[2, 3]. Therefore, it is an urgent need to study the carcinogenesis and progression of bladder cancer.

In recent years, accumulating data showed that epigenetic alterations play a significant role in carcinogenesis and progression of malignancies[4, 5]. Long noncoding RNAs (lncRNAs, >200nt) are the major noncoding RNAs that regulate gene expression at epigenetic, transcriptional, and post-transcriptional levels[6]. Long intervening noncoding RNAs (lincRNAs), as a subtype of lncRNAs, are transcript units discretely intervening known protein-coding loci. The important functions of lincRNAs have being demonstrated in many publications[7, 8]. Studies showed that abnormal expression of lincRNA is in a disease-, tissue- or developmental stage-specific manner[9, 10]. Some lincRNAs are involved in pathological processes of cancer, such as proliferation, apoptosis, invasion and metastasis, acting as tumor suppressor genes or oncogenes in various types of cancer[11, 12]. Nevertheless, there are thousands of functional lincRNAs yet to be identified. The objective of our study is to elucidate the lincRNAs expression patterns between bladder cancer and normal bladder tissues and try to find a specific lincRNA that may be a potential therapeutic target or biomarker for bladder cancer.

Studies have revealed that some aberrant expression of lincRNAs is closely associated with the progression and prognosis of bladder cancer, such as UCA1 and H19[13,14]. Our research team previously reported that linc-UBC1 played an important role in tumorigenesis and lymph node metastasis of bladder cancer[15]. In this report, we identified another novel lincRNA, termed as AATBC (Apoptosis-Associated Transcript in Bladder Cancer, also known as LOC284837). We found that AATBC was overexpressed in bladder cancer tissues and cancer cell lines. AATBC expression was positively correlated with tumor grade and pT stage of bladder cancer. AATBC knockdown resulted in inhibition of proliferation and increased apoptosis *in vitro* and suppressed tumor growth *in vivo*. The JNK signaling, as an important component of the mitogen-activated protein kinase (MAPK) signal transduction pathway[16], was activated by knockdown of AATBC. In addition, we found that NRF2, which is a critical player in the defense system against diverse stress condition including oxidative stress, was down regulated [17]. Inhibition of JNK by SP600125, ameliorated the cytotoxicity of AATBC silencing and resulted in up regulation of NRF2. These results indicated the key roles of JNK and NRF2 signaling pathway in the occurrence of apoptosis resulted from AATBC knockdown. Taken together, our results demonstrated that AATBC might play a critical role in promoting the tumorigenesis and progression of bladder cancer through the regulation of proliferation and apoptosis.

## RESULTS

### LincRNAs expression profiles in bladder cancer

**Figure 1:**
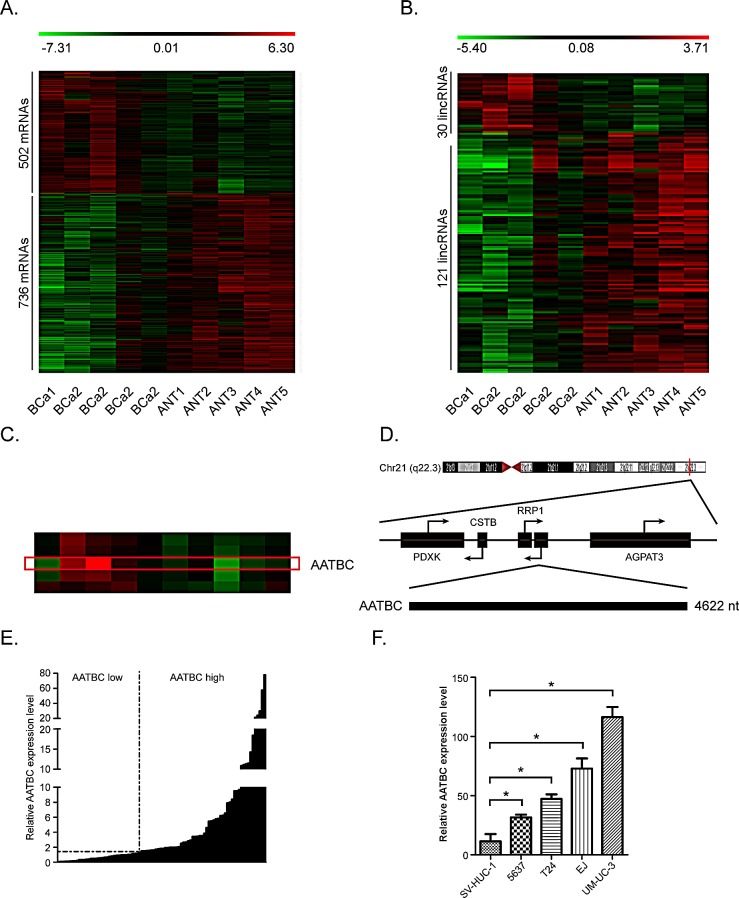
LincRNAs are dysregulated in bladder cancer A. Heat map representing unsupervised hierarchical clustering of mRNAs expression level in bladder cancer (BCa) tissues compared with adjacent non tumor (ANT) tissues. Each column represents the indicated tissue sample, and each row indicates one mRNA. Red color scale represents higher expression level and green color represents lower expression level. B. Heat map representing unsupervised hierarchical clustering of lincRNAs expression level in BCa tissues compared with ANT tissues. C. High resolution of the heat map showing AATBC expression in the microarray analysis. The red rectangular indicates the hybridization signal (replicate probes) of AATBC in BCa and ANT tissues. D. Genomic location of AATBC and its neighboring protein coding genes. E. Quantitative RT-PCR analysis of AATBC expression level in 90 cases of BCa relative to ANT tissues. Fold change of >1.5 is defined as overexpression (AATBC high), and the rest is indicated as AATBC low. F. The AATBC expression level in bladder cancer cell lines (UM-UC-3, T24, EJ, 5637) is higher than normal urothelium cell line SV-HUC-1.

To investigate the potential biological functions of lincRNAs in bladder cancer, we firstly examined the expression pattern (fold changes ≥ 2 and ≤0.5, *p* < 0.05) of lincRNAs and mRNAs in matched sets of muscle invasive bladder cancer tissues and adjacent non tumor tissues obtained from 5 patients. We used a microarray targeting 1238 Entrez protein coding genes and 151 lincRNAs (Agilent), as previously reported[15]. Hierarchical clustering was applied to analyze the systematic variations of lincRNAs expression in these 5 paired tissue specimens. Altogether, 30 lincRNAs were found to be up regulated more than two-fold, while 121 lincRNAs were down regulated more than two-fold (p < 0.05, Fig. 1A–B) in the bladder cancer tissues compared to the adjacent non tumor tissues. The microarray analysis of the expression pattern of lincRNAs clearly implicated that many lincRNAs are linked with bladder cancer. Some of these lincRNAs may have potential functions in the regulation of tumorigenesis and progression of bladder cancer or serve as molecular biomarkers.

### AATBC is overexpressed in bladder cancer

Then, we attempted to identify some lincRNAs that are overexpressed in bladder cancer. Firstly, we chose the first ten most overexpressed lincRNAs in the microarray analysis. Secondly we examined the expression levels of these lincRNAs in the bladder cancer tissues of 30 independent cases. Finally, we characterized the most frequently overexpressed lincRNA LOC284837 (we named it AATBC) in bladder cancer tissues compared to the adjacent non tumor tissues (Fig. 1C).

Information from UCSC Browser shows that AATBC is a transcript of 4622bp and localizes in human chromosome 21q22.3, 8465bp downstream of RRP1 gene and 38902bp upstream of CSTB gene (Fig. 1D). TESTCODE tool (http://www.genomicsplace.com/testcode.html) was employed to predict whether AATBC is likely to code for a protein. A score 0.4502 was obtained, suggesting that AATBC is probably a noncoding RNA.

We further examined its expression levels in bladder cancer tissues of 90 independent cases through quantitative RT-PCR. Compared with the non-tumor counterparts, it was found that AATBC was up-regulated (fold change of ≥1.5) in 54 cases (60%), whereas 36 cases (40%) were down-regulated or without obvious changes (Fig. 1E). To evaluate the significance of AATBC high expression in bladder cancer, we investigated the relationship between AATBC and clinical characteristics of patients (Table. 1). Overall, it was illustrated that the expression of AATBC was higher in MIBC (T2-T4) compared to that in NMIBC (Ta, Tis, T1), and positively correlated with the tumor grade, which indicated that the high expression level of AATBC was associated with tumor development and aggressiveness. However, we found no significant correlation between AATBC expression with gender, age and lymph node status.

We further verified the expression pattern of AATBC in bladder cancer cell lines (T24, EJ, UM-UC-3 and 5637) and an immortalized normal urothelium cell line SV-HUC-1 by quantitative RT-PCR, using β-actin as a reference gene. Similar to the alterations of paired tissue samples, quantitative RT-PCR showed the expression level of AATBC was significantly higher in bladder cancer cell lines than that in the normal urothelium cell (Fig. 1F), which confirmed that the expression of AATBC was correlated with malignancy. We speculated that AATBC could be a potential oncogene in bladder cancer.

**Table 1:** Characteristics of bladder cancer patients

Characteristics	Patients frequency (%)	AATBC		Chi-square	*p* value
		Low	High		
Total	90	36(40%)	54(60%)		
Gender					
Male	78 (86.7%)	31	47	0.016	0.899
Female	12 (13.3%)	5	7		
Age (yr)					
<65	44 (48.9%)	18	26	0.03	0.863
≥65	46 (51.1%)	18	28		
pT stage					
Ta, Tis, T1	41 (45.6%)	22	19	5.893	0.015
T2-T4	49 (54.4%)	14	35		
Tumor grade					
Low grade	16 (17.8%)	11	5	6.608	0.010
High grade	74 (82.2%)	25	49		
Lymph node status					
N0	17 (18.9%)	31	42	1.009	0.315
N1, N2	73 (81.1%)	5	12		

### AATBC knockdown inhibits proliferation of bladder cancer cells via cell cycle arrest

**Figure 2:**
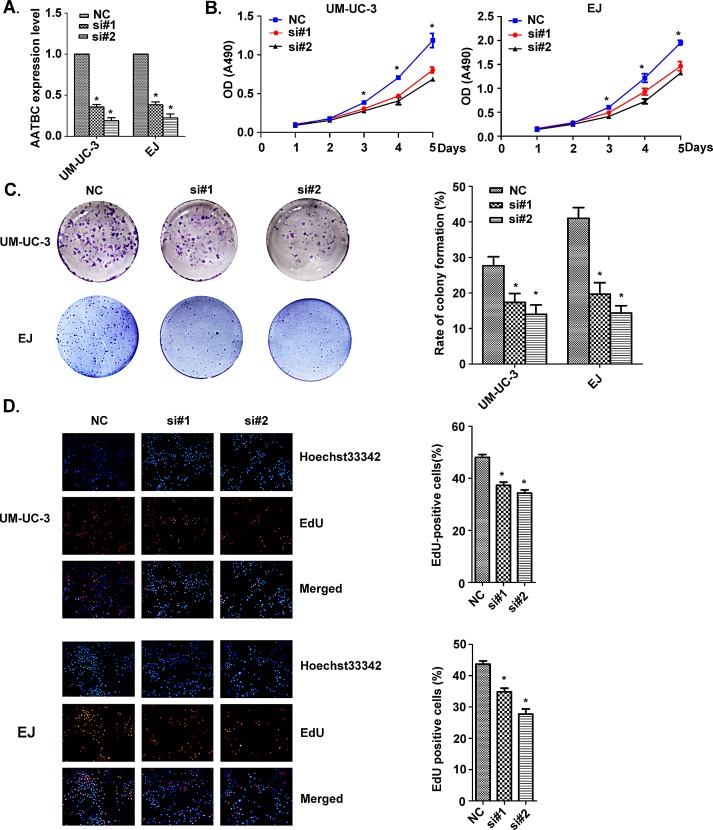
AATBC knockdown attenuated bladder cancer cell proliferation A. AATBC expression level was evaluated by quantitative RT-PCR in UM-UC-3 and EJ bladder cancer cell lines after transfection with siRNAs and negative controls. Means ± SD were shown (n = 3). *P<0.05 (vs. control). B. AATBC knockdown attenuated bladder cancer cell lines UM-UC-3 and EJ proliferation as determined by CellTiter 96 AQueous One Solution Cell Proliferation Assay. Means ± SD were shown (n = 3). *P<0.05 (vs. control). C. Colony formation by AATBC knockdown bladder cancer cells. Histological analysis of the rate of colony formation in control and AATBC knockdown groups. Means ± SD were shown (n = 3). *P<0.05 (vs. control). D. Analysis of the percent of EdU positive cells in negative control and AATBC knockdown groups in UM-UC-3 and EJ cells. Means ± SD were shown (n = 3). *P<0.05 (vs. control). AATBC knockdown led to decrease in S phase in both UM-UC-3 and EJ cells. Blue color represented the nucleus and red color indicated S phase cells (EdU positive).

We knocked down AATBC in UM-UC-3 and EJ bladder cancer cells using small interfering RNAs (siRNAs). The knockdown efficiency in UM-UC-3 cells was si#1 64.3% ± 3.0% and si#2 81.0% ± 3.6%. In EJ cells, the knockdown efficiency was si#1 61.7% ± 3.5% and si#2 77.7% ± 4.9% (Fig. 2A). In order to investigate the effect of AATBC on cell growth of bladder cancer cell lines *in vitro*, CellTiter 96 AQueous One Solution Cell Proliferation assay, panel clonogenic assay, EdU incorporation assay and flow cytometry were employed. These results show that AATBC depletion led to an obvious inhibitory effect on the growth of bladder cancer cells.

As shown in Fig. 2B, siRNAs-mediated knockdown of AATBC impaired the proliferation in UM-UC-3 and EJ cells, as revealed by CellTiter 96 AQueous One Solution Cell Proliferation assay. The number of live cells transfected with siRNAs was significantly decreased when compared with that in the negative controls. Consistent with the above results, the ability to form colonies of both cell lines was also suppressed significantly after down-regulation of AATBC when compared with that in the negative controls (Fig. 2C).

**Figure 3:**
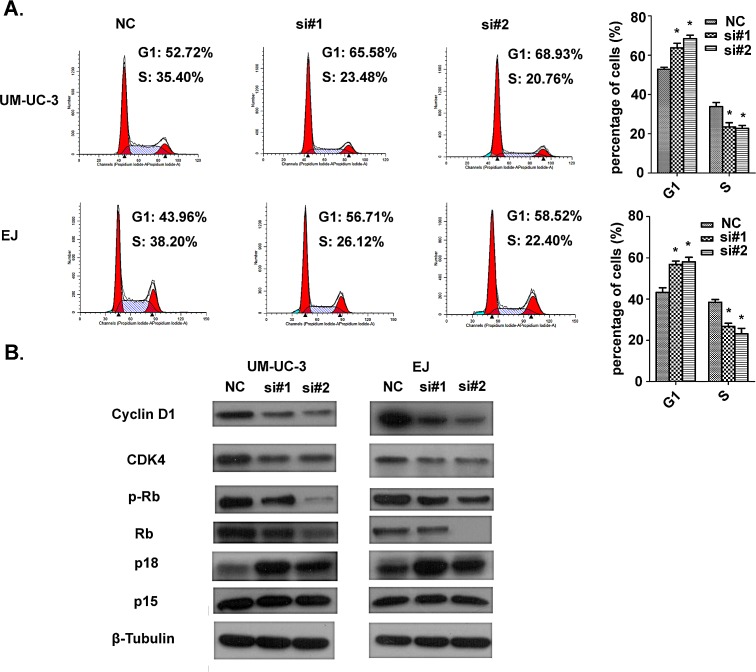
Effect of AATBC knockdown on cell cycle arrest A. Flow cytometric analysis of cell cycle arrest 48 hours after treatment of siRNAs and negative controls in UM-UC-3 and EJ cells. Data represented as means ± SD (n = 3). *P < 0.05 (vs. control). B. Western blotting analysis of the expression of cell cycle-related proteins.

For the purpose of better understanding the role of AATBC in proliferation, we employed the EdU incorporation assay to examine the effects of AATBC inhibition on the DNA synthesis during cell growth. The result showed that the proportion of S-phase cells (EdU-positive cells) was decreased in siRNA treated groups, suggesting reduced DNA synthetic activity resulted from AATBC depletion (Fig. 2D). Furthermore, we transfected the cancer cells with siRNAs before analyzing the impact on the cell cycle distribution by flow cytometry. Both UM-UC-3 and EJ cells treated with siRNAs show apparent increases in the percentage of cells in G1 phase with concomitant decreases in the percentage of cells in the S phase when compared with negative controls (Fig. 3A), which was consistent with EdU assay. These results proved that AATBC knockdown could lead to a cell cycle arrest in G1 phase, which was responsible for the suppressed proliferation.

To elucidate the possible molecular mechanisms accounting for the anticancer behaviors in proliferation triggered by AATBC depletion, we measured the expression of cell cycle-related proteins by Western blotting. Consistent with the observed phenomena of cell growth, the knockdown of AATBC led to an increased expression of p18 and decreased expression of cyclin D1, cyclin-dependent kinase 4 (CDK4) and phosphorylated retinoblastoma (Rb), without any obvious effect on p15 expression (Fig. 3B). In particular, changes of these cell cycle regulatory factors were correlated well with the G1 cell cycle arrest as shown in flow cytometry and EdU incorporation assay. Therefore, these data suggested that AATBC might be capable of promoting cell proliferation in bladder cancer through regulating cell cycle.

### AATBC knockdown inhibits tumor growth in NOD/SCID mice

**Figure 4:**
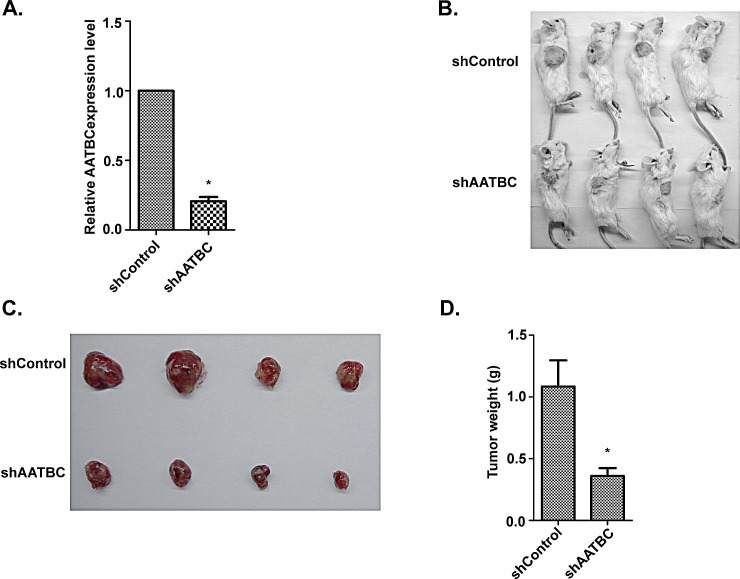
AATBC knockdown inhibited tumorigenesis by bladder cancer cells *in vivo* A. AATBC expression level was evaluated by quantitative RT-PCR in UM-UC-3 after transfection with shRNAs and negative controls. Means ± SD were shown (n = 3) *P < 0.05 (vs. control). B-C. Images of tumorigenesis assay performed in NOD/SCID mice. D. Histological analysis of tumor weight in control and AATBC stable knockdown group (Student's t-test, *P*<0.05). Means ± SD were shown (n = 4). * *P* < 0.05 (vs. control).

To investigate the biological significance of AATBC knockdown on tumor growth *in vivo*, we proceeded with the research through the establishment of a subcutaneous xenograft tumor model in NOD/SCID mice. Firstly, Stable AATBC-knockdown in UM-UC-3 cells by lentiviral-mediated RNAi system was successfully established. Quantitative RT-PCR analysis confirmed that the lentivirus-transduced UM-UC-3 cells showed a significant decrease of AATBC in transcript levels. The inhibition efficiency was 79.3% ± 3.1% (Fig. 4A). Equal numbers of UM-UC-3 cells (5×10^6^, per mice) stably expressing AATBC shRNA and negative control were injected subcutaneously into the flanks of NOD/SCID mice (n=4) (Fig. 4B). As expected, suppression of AATBC expression in UM-UC-3 cells treated with shRNA resulted in lower mean tumor mass compared to that of negative control group (0.36 g vs 1.085 g, P<0.05, Fig. 4C-D). The results *in vivo* indicated that the growth of xenografts was inhibited by AATBC shRNA treatment in UM-UC-3. This response of tumors to AATBC stable depletion revealed that AATBC might have tumor-promoting property in the carcinogenesis and progression of bladder cancer *in vivo*.

### Knockdown of AATBC results in the intrinsic apoptosis in bladder cancer cells

**Figure 5:**
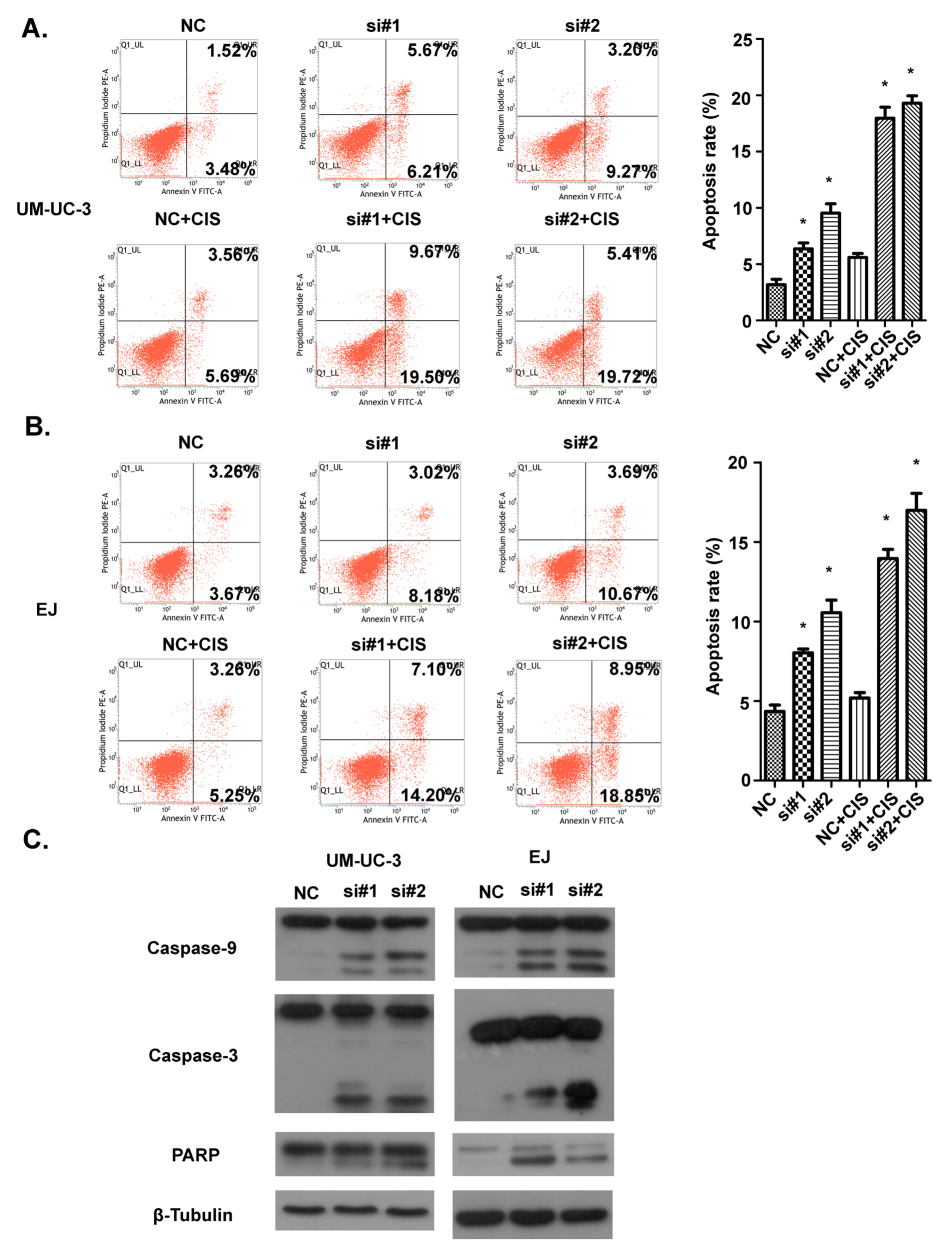
Effect of AATBC knockdown on bladder cancer apoptosis A. 48 hours after transfection, apoptosis rate in UM-UC-3 cells was analyzed by flow cytometry. In cisplatin-induced groups, cisplatin was added 24 hours before examination. Data represented as means ± SD. *P < 0.05 (vs. control). B. 48 hours after transfection, apoptotic rate in EJ cells was analyzed by flow cytometry. In cisplatin-induced groups, cisplatin was added 24 hours before examination. Data represented as means ± SD.*P < 0.05 (vs. control). C. Western blotting was used to detect the expression of caspase-9, caspase-3 and PARP. β-tubulin served as loading control.

We then investigated the involvement of AATBC in cell death of bladder cancer cells through inhibiting AATBC expression with siRNAs. As shown by flow cytometry analysis in Fig. 5A-B, siRNAs treatment led to the increased apoptotic rates in UM-UC-3 and EJ cells compared with that in negative control groups. After cells were induced with cisplatin, the pro-apoptotic effect of AATBC inhibition was augmented significantly when compared to that of no cisplatin-induced groups. Western blotting was used to investigate the alteration of apoptosis-related proteins induced by AATBC down-regulation. To our knowledge, the cleavages of caspase-9 and caspase-3 are prominent markers of the intrinsic apoptosis. In this present study, the occurrence and intensity of apoptosis resulted from AATBC knockdown were evidenced by the enhanced cleavages of poly (ADP-ribose) polymerase (PARP), caspase-9 and caspase-3 in both UM-UC-3 and EJ cell lines (Fig. 5C), indicating that the activation of intrinsic apoptotic pathway was involved in the cell apoptosis due to AATBC down regulation.

### AATBC knockdown induces activation of JNK signaling and suppression of NRF2

**Figure 6:**
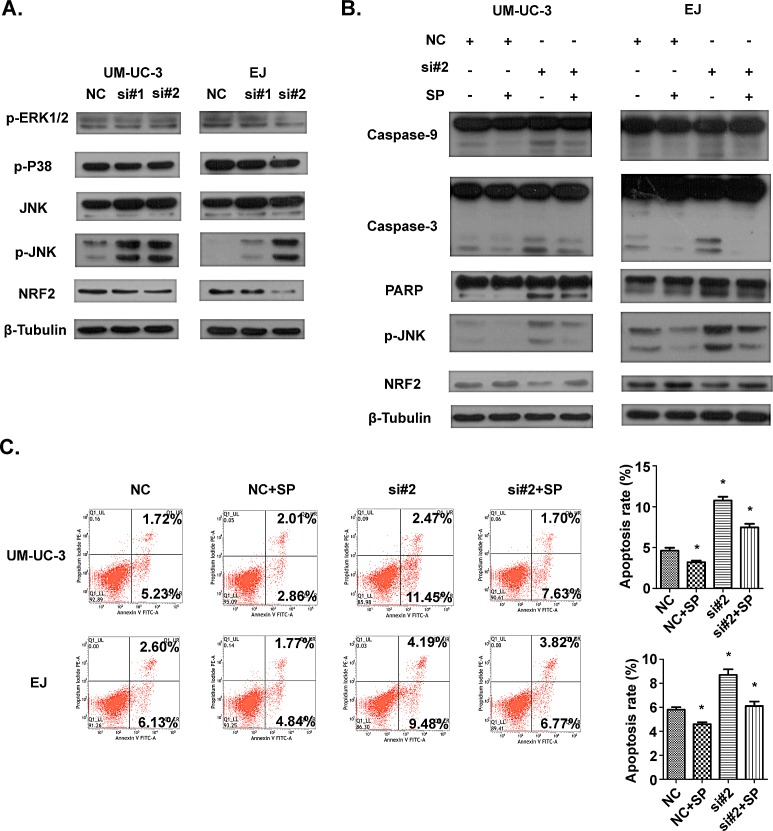
The involvement of JNK and NRF2 pathway in apoptosis induced by AATBC knockdown A. 36 hours after transfection, levels of JNK, p-JNK, p-p38, p-ERK1/2 and NRF2 were determined by Western blotting analysis. β-tubulin served as loading control. B. Indicated cell lines were treated with transfection and SP600125 for 36 hours. The levels of p-JNK, NRF2, caspase-9, caspase-3 and PARP were evaluated. β-tubulin served as loading control. C. 36 hours after treatment of transfection and SP600125, flow cytometric analysis showed that JNK inhibitor SP600125 could protect UM-UC-3 and EJ cells from apoptosis. Data was represented as means ± SD. *P <0.05 (vs. control).

In view of the evidence that the MAPKs, consisting of ERK1/2, JNK and p38, play critical roles in apoptosis, we examined the activation of MAPK pathway to further uncover the underlying mechanisms for the alterations of apoptosis in UM-UC-3 and EJ cells after transfection with siRNAs. As revealed by Western blotting (Fig. 6A), knockdown of AATBC enhanced the phosphorylation level of JNK in both UM-UC-3 and EJ cells. In contrast, the expression levels of phosphorylated ERK1/2 and p38 were not obviously changed upon the treatment of AATBC knockdown. JNK-mediated apoptosis can occur through multiple pathways, including oxidative stress (OS) pathway [18]. Inspired by this, we detected the factors in OS pathway. In this study, besides the activation of JNK, we found that the expression level of NRF2, a key antioxidant transcription factor in OS pathway, was down-regulated (Fig. 6A), indicating the impaired cellular self-protective response in bladder cancer cells after AATBC depletion. Furthermore, the treatment with the inhibitor of JNK (SP600125) could enhance the expression level of NRF2 (Fig. 6B) and improve the cell adaptive ability to compensate the stress condition, indicating a potential link between JNK activation and NRF2 inhibition. Collectively, these results demonstrated that apoptosis after AATBC knockdown might be mediated by the activation of JNK signaling and suppression of NRF2.

### Apoptosis induced by AATBC knockdown is rescued by inhibition of JNK signaling

To verify in depth whether this apoptotic phenomenon was dependent on the activation of JNK signaling pathway, the JNK-specific inhibitor SP600125 was supplemented to block JNK signaling before transfection with siRNAs to see whether the pro-apoptotic effect of AATBC down-regulation could be attenuated. As a result, Western blotting analysis demonstrated that SP600125 reduced the levels of the cleavage fragments of caspase-9, caspase-3 and PARP, and phosphorylated JNK efficiently in UM-UC-3 and EJ cells (Fig. 6B). In addition, it was also found by flow cytometry using Annexin V-FITC/PI staining (Fig. 6C) that SP600125 was able to decrease the percentage of apoptotic cells resulted from siRNA-mediated AATBC down regulation in comparison with the negative controls. These data further illustrated that the apoptosis induced by AATBC depletion in bladder cancer was mediated through the activation of JNK signaling.

## DISCUSSION

Because of the unlimited proliferation and apoptosis defect of cancer cells, treatment of cancer is still a huge challenge for human beings. In recent years, increasing studies have revealed that some lincRNAs appear to be correlated with aberrant proliferation and apoptosis of cancer cells, which may in part explain the critical roles of lincRNAs in cancer etiology[19,20]. For example, lncRNA HOXA-AS2 suppresses apoptosis in promyelocytic leukemia. Knockdown of HOXA-AS2 by shRNA resulted in an increase in ATRA-induced apoptosis through the cleavage of caspase-9, caspase-8 and caspase-3[21]. However, for most of these lincRNAs, the detailed functions, mechanisms and signaling pathways through which lincRNAs perform their biological functions have not been well understood. Studies focused on the regulatory functions and mechanisms of lincRNAs in bladder cancer are yet limited at present. Therefore, we conducted some researches to clarify the possible relationships between bladder cancer and lincRNAs and explore the potential application of lincRNAs in the diagnosis and treatment of bladder cancer.

The expression profiles of lincRNAs detected by microarray analysis provided valuable insight for the elucidation of functional roles and the underlying mechanisms of certain lincRNA in human cancer. In our study, we investigated the aberrant lincRNAs expression profiles in bladder cancer tissues by microarray analysis and identified a new lincRNA AATBC. We found that AATBC was overexpressed in bladder cancer tissues compared with adjacent non tumor tissues and positively correlated with tumor grade and pT stage, implying that AATBC might be an useful diagnostic biomarker or therapeutic target in bladder cancer[22]. This phenomenon was further demonstrated by the relative changes of biological behaviors in bladder cancer cell lines after AATBC knockdown. As mentioned above, down regulation of AATBC resulted in effectively suppressed proliferation *in vitro*, concomitant with induction of cell cycle arrest and apoptosis, and inhibited tumor growth in NOD/SCID mice *in vivo*. However, it remains unclear with respect to the relationship between AATBC expression levels and the overall survival of patients. This may be due to the limited number of cases and follow-up time. An extensive prospective study in larger cohorts may be needed in the future.

Cancer progression is often due to disorders in cell cycle that leads to the unlimited proliferation of cancer cells [23,24]. The cell cycle transition from the G1 phase to the S phase is the major regulatory checkpoint in this process. This transition is characterized by the phosphorylation of Rb and the interaction of CDKs, cyclins and CDK inhibitors (CDKI). In our study, the flow cytometry analysis and EdU incorporation assay demonstrated that AATBC down-regulation induced cell cycle arrest at the G1 phase and lower percentage of cancer cells in S phase. We also provided molecular evidences supporting the observed proliferation alterations after AATBC knockdown through evaluating the expression of proteins that play crucial role in G1 phase and the G1/S transition of the cell cycle. We found that knockdown of AATBC inhibited the cyclin D1, CDK4 and p-Rb accompanied by a decrease in total Rb, with an increasing expression of p18. To our knowledge, cyclin D1, CDK4 and Rb play important roles in this process [25]. Cyclin D1 promotes cells to go through the G1 phase via activating CDK4, which leads to increased phosphorylation of Rb (p-Rb) [26]. The Ink4 (Inhibitor of CDK4) family, such as p15 (INK4B) and p18 (INK4C), can specifically inhibit complex of CDK4-cyclin D and further decrease phosphorylation of Rb to regulate cell cycle[27]. Thus, the most likely mechanism underlying the growth arrest involves an increase in p18 expression, which leads to an inhibition of the complex of CDK4-cyclin D1 and phosphorylation of Rb, and then ultimately stimulate cell cycle arrest at the G1 phase. This cell cycle arrest was attributed to, at least in part, the anticancer effect of AATBC knockdown on proliferation. Collectively, these data shed light on a vital role of AATBC in promoting tumorigenesis and progression of bladder cancer. AATBC could be a potential therapeutic target for bladder cancer.

In the process of cell apoptosis, the caspase family is indispensable for the initiation and execution of cell death in response to various kinds of stimuli [28-30]. The intrinsic apoptotic signal can recruit and activate initiator caspase-9 and effector caspase (caspase3/6/7), which bring about cellular death ultimately. Our results showed that the knockdown of AATBC by RNAi technology led to the apoptosis of bladder cancer cells via the intrinsic apoptosis signal, as shown by the activation of caspase-9 and caspase-3. Moreover, cisplatin induction could significantly strengthen the pro-apoptotic ability of AATBC knockdown, indicating that apoptosis via AATBC inhibition could enhance the chemosensitivity of bladder cancer cells and AATBC might be an attractive therapeutic target in bladder cancer treatment.

The dysregulation of urothelial epithelial cell apoptosis is one of the causative factors of bladder cancer. Various factors may affect apoptosis of cancer cells through different signaling pathways [31,32]. JNK, as a serine-threonine kinase and a main member of the MAPK pathway, plays an important role in a variety of physiological and pathological processes, including cell death or apoptosis [33,34]. In this study, AATBC down-regulation modulated the phosphorylation level of JNK and exerted its influence on apoptosis in bladder cancer cells. The interplay between AATBC and JNK signaling was highlighted by using the JNK inhibitor SP600125, which attenuate the pro-apoptotic ability of AATBC knockdown.

The transcription factor NF-E2-related factor 2 (NRF2) plays a major role in the unfolded protein response (UPR). It has been accepted widely that NRF2 is important in the protective system against oxidative stress, electrophilic stress and the other adverse condition through transcriptional regulation of the genes encoding antioxidant proteins, detoxification enzymes and other stress response regulators [35,36]. Elevated expression of NRF2 can protect cancer cells from harm by chemotherapeutic drugs, including cisplatin, doxorubicin and etoposide [37]. On the contrary, NRF2-deficiency enhances the susceptibility of cancer cells to hostile stimuli [38]. We showed for the first time that AATBC knockdown resulted in decreased level of NRF2, which might partly contribute to the higher apoptotic rates after adding of cisplatin with siRNAs compared to the no cisplatin-induced groups. Although our findings imply a molecular mechanism by which oxidative stress may involve in the apoptosis resulting from AATBC inhibition, further investigation is required to document this in the future.

Therefore, the pro-apoptotic effect of AATBC knockdown in bladder cancer may achieved through activation of JNK signaling and suppression of NRF2. AATBC may be an important regulator of apoptosis in bladder cancer. Its overexpression hiders the apoptosis of tumor cells and increases the number of tumor cells, thereby promoting tumor growth, which partially explain AATBC may serve as a potential oncogene in bladder cancer.

To conclude, the data presented in our study collectively provides convincing evidences that AATBC exhibits potentially pro-proliferative and anti-apoptotic functions in bladder cancer. The most possible mechanisms underlying these functions include disregulation in the cell cycle, activity of the intrinsic apoptosis that involved in JNK and NRF2 signaling pathway. Both *in vitro* and *in vivo* results strengthened the positive links between AATBC expression and clinical parameters, including tumor grading and pT stage. Therefore, AATBC could be a promising therapeutic target and molecular biomarker for bladder cancer.

## MATERIALS AND METHODS

### Microarray and computational analysis

Total RNA from 5 pairs of bladder cancer with matched normal tissue specimens was extracted and purified by RNeasy mini kit (QIAGEN) with RNase-free DNase set (QIAGEN). These total RNAs were then amplified and labeled by Low Input Quick Amp Labeling Kit, One-Color (Agilent), according to the manufacturer's protocols. Each Slide was hybridized with Cy3-labeled cRNA using Gene Expression Hybridization Kit (Agilent). After hybridization, slides were washed in staining dishes (Thermo Shandon) with Gene Expression Wash Buffer Kit (Agilent), following the manufacturer's instructions. Slides were scanned by Agilent Microarray Scanner (Agilent). The acquired array images were analyzed with Agilent Feature Extraction software (version 11.0.1.1). Quantile normalization and subsequent data processing were performed using the Gene Spring Software 11.0 (Agilent). The microarray work was performed by KangChen Bio-tech, Shanghai, China.

### Patients and tissue samples of bladder cancer

Ninety paired samples of bladder cancer and adjacent non tumor tissues (2 cm away from the tumor) were obtained from patients, who received cystectomy in Sun Yat-sen Memorial Hospital of Sun Yat-sen University. After resection, all samples were immersed immediately in RNAlater solution (Ambion, Austin, Texas) overnight, then stored at −80°C in order to avoid degradation of RNA. All the bladder cancer patients were graded according to the WHO grading system (low-grade, 16 cases; high-grade, 74 cases) and were staged according to the TNM classification (NMIBC Tis, Ta, T1: 41 cases; MIBC >T1: 49 cases). Prior to the use of these clinical materials for research purposes, written consents from all patients and approval of the Hospital Ethic Review Committees were obtained.

### Cell culture

The human bladder cancer cell lines (UM-UC-3, T24 and 5637) and an immortalized normal urothelium cell line (SV-HUC-1) were purchased from the American Type Culture Collection (ATCC). Bladder cancer cell line EJ was obtained from the Institute of Biochemistry and Cell Biology, Shanghai Institutes for Biological Sciences, Chinese Academy of Sciences (Shanghai, China). EJ, T24 and 5637 cell lines were routinely cultivated in RPMI-1640 (Gibco) while UM-UC-3 cell line was grown in DMEM (Gibco), and SV-HUC-1 in F-12K medium (Gibco). All the media contain 10% fetal bovine serum (FBS) (Bioind), 100 U/mL penicillin and 100 μg /mL streptomycin (Gibco). Cells were cultured and maintained under standard conditions of 5% CO_2_ and 37°C.

### Antibodies and reagents

Antibodies, including anti-cyclin D1, CDK4, p15, p18, Rb, p-Rb, caspase-3, caspase-9, PARP, JNK, p-JNK, p-P38, p-ERK1/2, β-Tubulin and HRP-linked secondary antibody were obtained from Cell Signaling Technology. The anti-NRF2 antibody was purchased from Bioworld Technology. All the antibodies were diluted 1:1000. SP600125, as a JNK inhibitor, was purchased from Sigma. SP600125 was dissolved in DMSO as a 20 mmol/L stock solution and stored at −20°C. To avoid precipitation of SP600125, DMSO was added to a final concentration of 0.1% in the media. UM-UC-3 and EJ cells were treated with 5 μmol/L SP600125 1 hour before transfection with siRNAs. Cisplatin was obtained from the institutional pharmacy (Qilu Pharmaceutical Co., China). In the groups of cisplatin-induced apoptosis of tumor cells, cisplatin was added at concentration of 1.5 μg/ml to UM-UC-3 ells and μg/ml to EJ 3 cells, respectively.

### RNA extraction and quantitative RT-PCR

According to the manufacturer's instructions, total RNA from cell lines and tissue samples was extracted using Trizol reagent (Invitrogen). First-strand cDNA was synthesized with PrimeScript RT Master Mix (TAKARA, Dalian, China). After reverse transcription of the total RNA, quantitative RT-PCR was conducted to examine the expression of AATBC using SYBR-Green PCR Master mix (Roche) on LightCycler 480 Real-Time PCR instrument (Roche). β-actin was used as an internal reference gene to normalize RNA levels between different samples for an exact comparison of transcription level. The sequences of primers were as follows (in the 5′ to 3′ orientation): β-actin forward, ACTGGAACGGTGAAGGTGAC, β-actin reverse, AGAGAAGTGGGGTGGCTTTT; AATBC forward, AAGGCCGGTTATCAACGT, AATBC reverse, GCCAGTCCCTCACTGCTCT. Relative quantitative expression of AATBC was determined using the ΔΔCT method and presented as relative units.

### Knockdown of AATBC expression

In the transient transfection experiments, the siRNAs targeting AATBC (si#1: CCAUGCACGGAUCUGACUUTT, AAGUCAGAUCCGUGCAUGGTT. si#2: CGGUCAUAUUUGAGCAUGATT, UCAUGCUCAAAUAUGACCGTT) and the negative control siRNA (UUCUCCGAACGUGUCACGUTT, ACGUGACACGUUCGGAGAATT) were purchased from GenePharma (Shanghai, China). UM-UC-3 and EJ cells were seeded at a density of 1.5×10^5^ cells per well in 6-well plates. Twenty-four hours later, cells were transfected with siRNAs using Lipofectamine RNAiMAX Transfection Reagent (Life technologies) following manufacturer's instructions. siRNA knockdown was assessed by quantitative RT-PCR.

In the animal experiment, for stable knockdown of AATBC in UM-UC-3 cells, we constructed lentiviruses containing AATBC shRNA, which was synthesized by Shanghai GeneChem Co. Ltd (Shanghai, China). The short hairpin RNAs (shRNAs) were cloned into the GV248-GFP lentiviral vector. The sh-AATBC targeting sequence was CAGATACACTGACTACGAT. A nontargeting scrambled shRNA GV248-GFP vector was generated as a negative control. These modified plasmids were further co-transfected into HEK 293T cells with lentiviral packaging plasmids to generate an AATBC shRNA-expressing lentivirus or a control shRNA-expressing lentivirus. For cell infection, UM-UC-3 cells were cultured in 6-well plates at a density of 3 × 10^5^ cells per well and infected with the constructed lentiviruses expressing AATBC shRNA and scrambled shRNA, respectively. Forty-eight hours after infection, 1 mg/ml puromycin (Invitrogen) was added into the media to select puromycin-resistant clones. The inhibitory efficiency of AATBC was detected by quantitative RT-PCR.

### 5-ethynyl-2′-deoxyuridine (EdU) incorporation assay

UM-UC-3 and EJ cells (1.0 × 10^4^ cells/well) were seeded into 24-well culture plates, followed by transfection with siRNAs to knockdown AATBC expression. Forty-eight hours after transfection, cell proportion in S-phase was analyzed by EdU assay (Cell-Light™ EdU DNA Cell Proliferation Kit, Ribobio, Guangzhou, China) according to the manufacturer's instructions. The procedures were as follows: cells were incubated in complete medium supplemented with 50 μM EdU for 2 hours at 37 °C, washed with phosphate-buffered saline (PBS) and then cells were fixed with 4% paraformaldehyde for 30 minutes at room temperature. The excess paraformaldehyde was neutralized with 2 mg/ml glycine. After being washed with PBS and 0.5% Triton X-100 in PBS, cells were washed with PBS again and dyed with 1× Apollo® reaction cocktail in dark for 30 minutes. Cells were washed twice with 0.5% Triton X-100 in PBS again, then incubated with 1× Hoechst 33342 solution for 30 minutes in dark at room temperature. At the end, after washing with PBS for three times, cellular immunostaining was observed with fluorescence microscopy and photographed. Digital images were acquired and analyzed with Image J software.

### CellTiter 96 Aqueous One Solution Cell Proliferation assay

The effect of AATBC knockdown on cell growth of UM-UC-3 and EJ was measured using CellTiter 96 Aqueous One Solution Cell Proliferation Assay kit (Promega). One day after treatment of siRNAs, cells were trypsinized and collected. 1000 cells per well were seeded in 96-well plates and allowed to attach overnight. From the second day, CellTiter 96 Aqueous One Solution was added in at least five replicate wells at one-fifth of the total volume and incubated for 4 hours at 37°C. Absorbance was measured with the multifunctional microplate reader SpectraMax M5 (Molecular Devices) at 490 nm. The measurement of cell proliferation was conducted every 24 hours, and lasted 5 days. Cell growth curve was constructed with absorbance as ordinate and time as abscissa.

### Colony formation assay

UM-UC-3 and EJ cells, treated by siRNAs and negative control for 24 hours, were routinely trypsinized and seeded in 6-well plates (1000 cells/well). The medium was changed every three days. After two weeks, cells were washed twice with PBS, fixed with 4% paraformaldehyde for 30 minutes, and then stained with crystal violet for 30 minutes for visualization and counting.

### Flow cytometry assay

Flow cytometry analysis was performed to determine whether AATBC regulates the growth phase and apoptosis of bladder cancer cells. UM-UC-3 and EJ cells were seeded into 6-well plates. After tightly attaching to sidewall of plates, cells were transfected with siRNAs. Forty-eight hours after transfection, the cells were harvested. In cisplatin-induced groups, cisplatin was added 24 hours before harvest. When evaluating the effect of JNK pathway blockage, the cells were pre-incubated with 5 μmol/L SP600125 for 1 hour, and then treated with siRNAs for 36 hours. The cells were stained with annexin V-FITC and propidium iodide (PI), according to the manufacturer's instructions. Cellular apoptotic rate was evaluated by FACSVerse^TM^ flow cytometer (Becton Dickinson, CA, USA). Cells for growth phase analysis were resuspended in 200 μl PBS, fixed with 70% ice-cold ethanol overnight, and stained with PI. The cell cycle was detected by FACSVerse^TM^ flow cytometer.

### Protein extraction and Western blotting

Cells were rinsed twice with cold PBS and lysed by RIPA buffer (Thermo) containing protease inhibitor cocktail (Roche). Total protein was extracted according to the manufacturer's instruction. The concentration of protein was quantitated by bicinchoninic acid method. For Western blotting analysis, fifty μg protein of each sample was separated by electrophoresis in 10%~12% sodium dodecylsulfate-polyacrylamide gel and was then transferred to polyvinylidene fluoride membranes (Millipore). Blotted membranes were blocked in 5% skimmed milk in TBST, followed by incubation with appropriate primary antibodies overnight at 4°C. Then, the membranes were washed 5 minutes for three times with TBST, and subsequently incubated 1 hour with HRP-linked secondary antibody at room temperature. β-Tubulin was used as an internal control. The blots were detected by enhanced chemiluminescence kit (Millipore) and autoradiography using X-ray film.

### Xenograft model in NOD/SCID mice

Male NOD/SCID mices (4 weeks, 14-16 g body weight) were raised under specific pathogen free (SPF) conditions. All the experimental procedures were performed according to the institutional ethical guidelines for animal experiment. For establishing bladder cancer xenograft model, UM-UC-3 cell line was used. Eight NOD/SCID mice were randomly divided into two groups: the lentiviral-mediated AATBC stable knockdown group (n=4) and the negative control group (n=4). Eight NOD/SCID mice were subcutaneously injected into the flank with UM-UC-3 cells at the dose of 5 ×10^6^ cells suspended in 200 μl PBS. Animals of both groups were sacrificed and tumor weight was measured after 4 weeks.

### Statistic analysis

All the experiments were performed at least three times, and then mean values and standard deviation were calculated. Differences between two groups were analyzed by Student's *t*-test. The relationship between AATBC and the other clinical characteristics was determined using SPSS 22.0 Pearson Chi-square test. A value of *P* < 0.05 was considered to be statistically significant.

## SUPPLEMENTARY MATERIAL AND FIGURES



## References

[1] Jemal A, Bray F, Center MM, Ferlay J, Ward E, Forman D (2011). Global cancer statistics. CA Cancer J Clin.

[2] Shariat SF, Lotan Y, Vickers A, Karakiewicz PI, Schmitz-Dräger BJ, Goebell PJ, Malats N (2010). Statistical consideration for clinical biomarker research in bladder cancer. Urologic Oncology: Seminars and Original Investigations.

[3] Parker J, Spiess PE (2011). Current and Emerging Bladder Cancer Urinary Biomarkers. The Scientific World JOURNAL.

[4] Neureiter D (2014). Epigenetics and pancreatic cancer: Pathophysiology and novel treatment aspects. World Journal of Gastroenterology.

[5] Kang C (2014). Epigenetics: An emerging player in gastric cancer. World Journal of Gastroenterology.

[6] Vance KW, Ponting CP (2014). Transcriptional regulatory functions of nuclear long noncoding RNAs. Trends in Genetics.

[7] Beckedorff Felipe C, Amaral Murilo S, Deocesano-Pereira C, Verjovski-Almeida S (2013). Long non-coding RNAs and their implications in cancer epigenetics. Bioscience Reports.

[8] Maruyama R, Suzuki H (2012). Long noncoding RNA involvement in cancer. BMB reports.

[9] Nam JW, Bartel DP (2012). Long noncoding RNAs in C. elegans. Genome Research.

[10] Pastori C, Wahlestedt C (2012). Involvement of long noncoding RNAs in diseases affecting the central nervous system. RNA Biology.

[11] Enfield KSS, Pikor LA, Martinez VD, Lam WL (2012). Mechanistic Roles of Noncoding RNAs in Lung Cancer Biology and Their Clinical Implications. Genetics Research International.

[12] Hao Li, Beiqin Yu, Jianfang Li, Liping Su, Min Yan, Zhenggang Zhu, Bingya Liu (2014). Overexpression of lncRNA H19 enhances carcinogenesis and metastasis of gastric cancer. Oncotarget.

[13] Wang F, Li X, Xie X, Zhao L, Chen W (2008). UCA1, a non-protein-coding RNA up-regulated in bladder carcinoma and embryo, influencing cell growth and promoting invasion. FEBS Lett.

[14] Luo M, Li Z, Wang W, Zeng Y, Liu Z, Qiu J (2013). Long non-coding RNA H19 increases bladder cancer metastasis by associating with EZH2 and inhibiting E-cadherin expression. Cancer Lett.

[15] He W, Cai Q, Sun F, Zhong G, Wang P, Liu H, Luo J, Yu H, Huang J, Lin T (2013). linc-UBC1 physically associates with polycomb repressive complex 2 (PRC2) and acts as a negative prognostic factor for lymph node metastasis and survival in bladder cancer. Biochimica et Biophysica Acta (BBA) - Molecular Basis of Disease.

[16] Davies C, Tournier C (2012). Exploring the function of the JNK (c-Jun N-terminal kinase) signalling pathway in physiological and pathological processes to design novel therapeutic strategies. Biochem Soc Trans.

[17] Namani A, Li Y, Wang XJ, Tang X (2014). Modulation of NRF2 signaling pathway by nuclear receptors: implications for cancer. Biochim Biophys Acta.

[18] Luo Z, Dong X, Ke Q, Duan Q, Shen L (2014). Chitooligosaccharides inhibit ethanol-induced oxidative stress via activation of Nrf2 and reduction of MAPK phosphorylation. Oncol Rep.

[19] De Ocesano-Pereira C, Amaral MS, Parreira KS, Ayupe AC, Jacysyn JF, Amarante-Mendes GP, Reis EM, Verjovski-Almeida S (2014). Long non-coding RNA INXS is a critical mediator of BCL-XS induced apoptosis. Nucleic Acids Research.

[20] Kim K, Jutooru I, Chadalapaka G, Johnson G, Frank J, Burghardt R, Kim S, Safe S (2012). HOTAIR is a negative prognostic factor and exhibits pro-oncogenic activity in pancreatic cancer. Oncogene.

[21] Zhao H, Zhang X, Frazão JB, Condino-Neto A, Newburger PE (2013). HOX antisense lincRNA HOXA-AS2 is an apoptosis repressor in all trans retinoic acid treated NB4 promyelocytic leukemia cells. Journal of Cellular Biochemistry.

[22] Zhang X, Gejman R, Mahta A, Zhong Y, Rice KA, Zhou Y, Cheunsuchon P, Louis DN, Klibanski A (2010). Maternally Expressed Gene 3, an Imprinted Noncoding RNA Gene, is Associated with Meningioma Pathogenesis and Progression. Cancer Research.

[23] Gomez D (2012). Telomere structure and telomerase in health and disease (Review). International Journal of Oncology.

[24] Xu C, Zeng Q, Xu W, Jiao L, Chen Y, Zhang Z, Wu C, Jin T, Pan A, Wei R, Yang B, Sun Y (2012). miRNA-100 Inhibits Human Bladder Urothelial Carcinogenesis by Directly Targeting mTOR. Molecular Cancer Therapeutics.

[25] Chiron D, Martin P, Di Liberto M, Huang X, Ely S, Lannutti BJ, Leonard JP, Mason CE, Chen-Kiang S (2013). Induction of prolonged early G1 arrest by CDK4/CDK6 inhibition reprograms lymphoma cells for durable PI3Kδ inhibition through PIK3IP1. Cell Cycle.

[26] Gogolin S, Ehemann V, Becker G, Brueckner LM, Dreidax D, Bannert S, Nolte I, Savelyeva L, Bell E, Westermann F (2013). CDK4 inhibition restores G_1_-S arrest in MYCN-amplified neuroblastoma cells in the context of doxorubicin-induced DNA damage. Cell Cycle.

[27] Matsuzaki Y, Sakai T (2005). INK4 Family - A promising target for gene-regulating chemoprevention and molecular-targeting prevention of cancer. Environ Health Prev Med.

[28] White E (1996). Life, death, and the pursuit of apoptosis. Genes & Development.

[29] Fiandaloand M.V., Kyprianou N (2012). Caspase control: protagonists of cancer cell apoptosis. Exp Oncol.

[30] Hensley P, Mishra M, Kyprianou N (2013). Targeting caspases in cancer therapeutics. Biological Chemistry.

[31] Huang C, Bruggeman LA, Hydo LM, Miller RT (2012). Shear stress induces cell apoptosis via a c-Src-phospholipase D-mTOR signaling pathway in cultured podocytes. Experimental Cell Research.

[32] Tanaka T, Nakatani T, Kamitani T (2012). Inhibition of NEDD8-conjugation pathway by novel molecules: Potential approaches to anticancer therapy. Molecular Oncology.

[33] Dhanasekaran DN, Reddy EP (2008). JNK signaling in apoptosis. Oncogene.

[34] Huang CY, Kuo WW, Yeh YL, Ho TJ, Lin JY, Lin DY, Chu CH, Tsai FJ, Tsai CH, Huang CY (2014). ANG II promotes IGF-IIR expression and cardiomyocyte apoptosis by inhibiting HSF1 via JNK activation and SIRT1 degradation. Cell Death Differ.

[35] Jaramillo MC, Zhang DD (2013). The emerging role of the Nrf2-Keap1 signaling pathway in cancer. Genes Dev.

[36] Shelton LM, Park BK, Copple IM (2013). Role of Nrf2 in protection against acute kidney injury. Kidney Int.

[37] Hayden A, Douglas J, Sommerlad M, Andrews L, Gould K, Hussain S, Thomas GJ, Packham G, Crabb SJ (2014). The Nrf2 transcription factor contributes to resistance to cisplatin in bladder cancer. Urol Oncol.

[38] Duong HQ, Yi YW, Kang HJ, Hong YB, Tang W, Wang A, Seong YS, Bae I (2014). Inhibition of NRF2 by PIK-75 augments sensitivity of pancreatic cancer cells to gemcitabine. Int J Oncol.

