# Supplementary material for: Knockdown of a novel lincRNA AATBC suppresses proliferation and induces apoptosis in bladder cancer
Source: Oncotarget. 2014 Nov 25;6(2):1064–78. doi: 10.18632/oncotarget.2833 (PMC4359217; doi:10.18632/oncotarget.2833)
Supplement: Supplementary file 1 [file oncotarget-06-1064-s001.pdf]

Knockdown of a novel LincRNA AATBC suppresses proliferation and induces apoptosis in bladder cancer

Supplementary Material

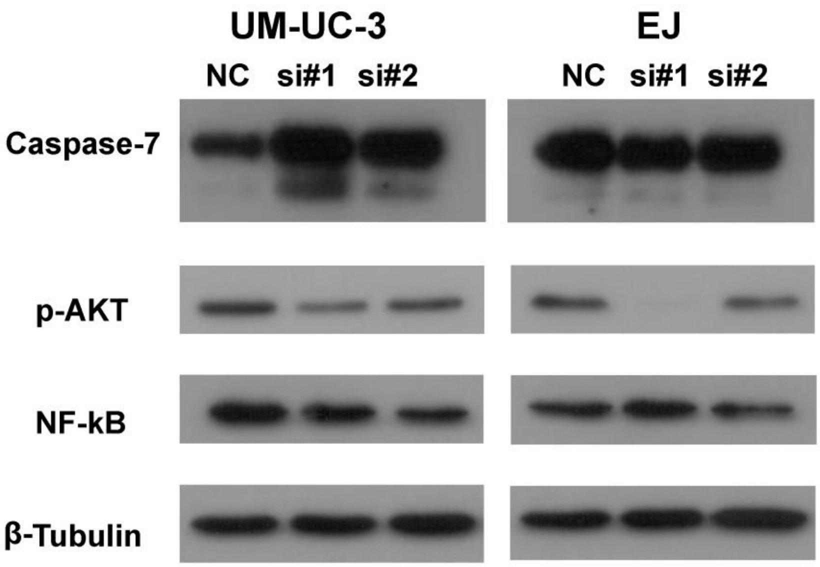

Supplementary Figure1

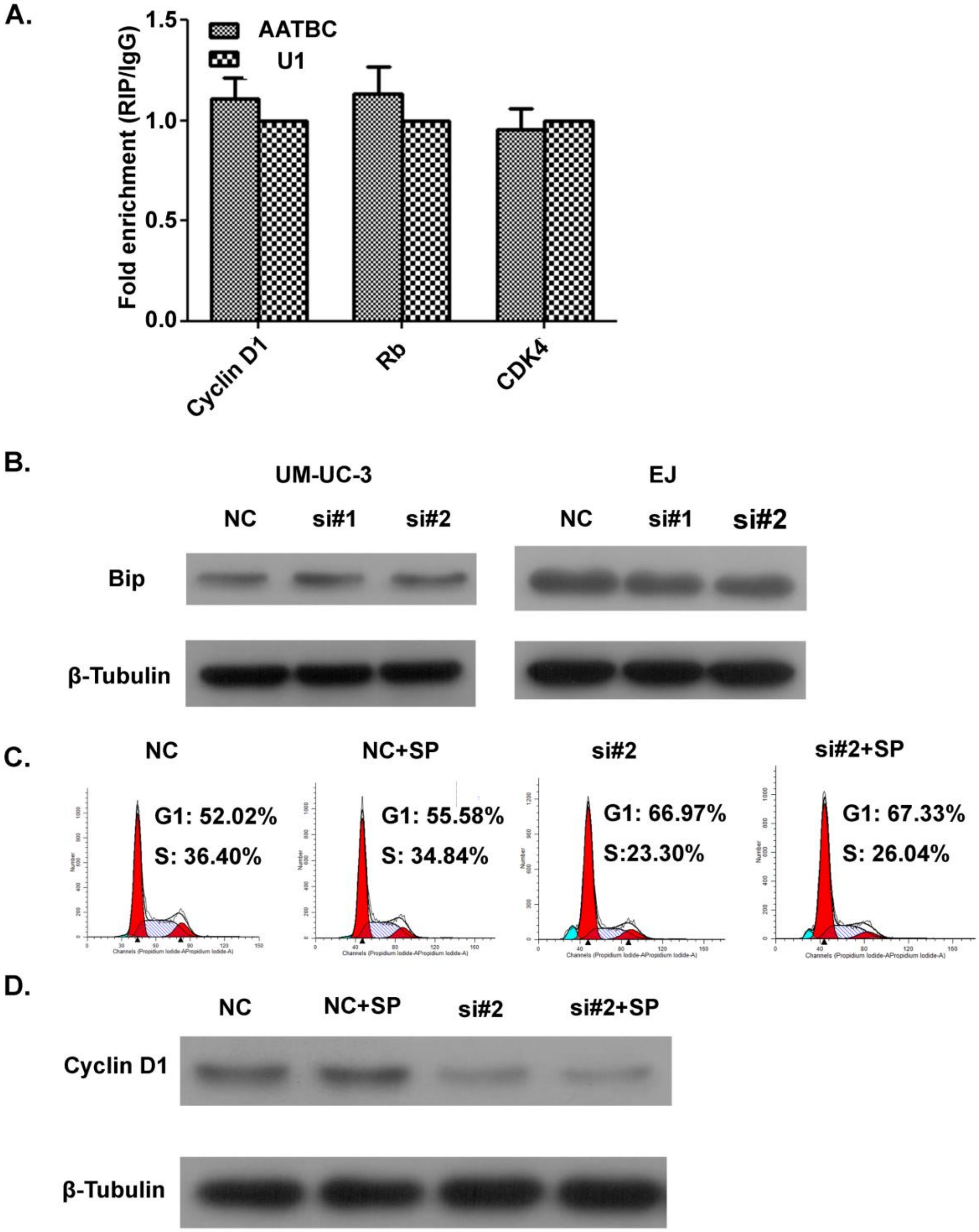

Supplementary Material 2
